# Human papillomavirus prevalence and associated factors in women and men in south China: a population-based study

**DOI:** 10.1038/emi.2016.118

**Published:** 2016-11-23

**Authors:** Feixue Wei, Kai Yin, Xin Wu, Jian Lan, Shoujie Huang, Wei Sheng, Jun Zhao, Yingying Su, Ying Wang, Yanping Li, Rongcheng Li, Jun Zhang, Mingqiang Li, Ting Wu, Ningshao Xia

**Affiliations:** 1 State Key Laboratory of Molecular Vaccinology and Molecular Diagnostics, National Institute of Diagnostics and Vaccine Development in Infectious Diseases, Strait Collaborative Innovation Center of Biomedicine and Pharmaceutics, School of Public Health, Xiamen University, Xiamen 361102, Fujian, China; 2 Liuzhou Center for Disease Control and Prevention, Liuzhou 545027, Guangxi, China; 3 Policy Coordination Division, China National Center for Biotechnology Development, Beijing 100039, China; 4 Centre for Vaccine Clinical Research, Guangxi Center for Disease Control and Prevention, Nanning 530028, Guangxi, China

**Keywords:** human papillomavirus, men, prevalence, risk factors, women

## Abstract

Oncogenic human papillomavirus (HPV) infection is a cause of many anogenital cancers in women and men; however, there is little research on HPV prevalence and risk factors that includes both women and men from the same population. A total of 4687 participants, including 2378 women and 2309 men aged 18–55 years old from the same community, were enrolled in the study in Liuzhou, China. Exfoliated cells were collected from the participants from different anatomic sites and were tested for 13 oncogenic and 3 non-oncogenic HPV types. The prevalence of any oncogenic HPV type was higher in women than in men (18.7% vs 9.4%, *P*<0.001), whereas the prevalence of HPV 6 and 11 infection was similar (1.4% vs 1.2%, *P*=0.6832). HPV 52, 58, 16, 39 and 18 were the five most prevalent types in both sexes. Sexual and hygienic behaviors were associated with HPV infection in both women and men. We found that oncogenic HPV DNA detection is more prevalent in women than in men in China, whereas the prevalence of HPV 6 and 11 is similar in both sexes. The data indicate that the interaction of host and virus might be different among high- and low-risk HPV types.

## INTRODUCTION

Oncogenic human papillomavirus (HPV) infection is a cause of many cancers in the anogenital and oropharyngeal areas in women and men. HPV is assumed to be responsible for 100% of cervical cancers, 90% of cancers of the anus, 50% of cancers of the penis, 40% of cancers of the vulva (VU), 70% of cancers of the vagina (VA) and 20%–60% of cancers of the oropharynx.^1^ Non-oncogenic HPV, especially HPV 6 and 11, is responsible for up to 90% of genital warts in both sexes.^2^

The first generation of preventive HPV vaccines are bivalent (HPV 16 and 18) or quadrivalent (HPV 6, 11, 16 and 18) and can prevent ~70% of cervical cancer cases. The second-generation HPV vaccine is nine-valent, adding five more HPV types (HPV 31, 33, 45, 52 and 58) to the quadrivalent HPV vaccine and is expected to prevent 20% more cervical cancer cases.^1^ Licensed HPV vaccines are used mainly in girls aged ≥nine years and in young women. HPV is mainly transmitted through sexual activity, and case–control studies have shown that the sexual behavior of male partners affects a woman's risk of cervical neoplasia.^3, 4^ Recently, a cohort study conducted in heterosexual couples illustrated that the male-to-female and female-to-male HPV transmission rates were 7.11 and 5.56 per 1000 person months, respectively.^5^ Therefore, the epidemiological characterization of HPV infection in both sexes from the same population is important to understand the whole picture of HPV transmission and natural history in humans.

Much has been learned about HPV prevalence in women and men, but few studies have included both sexes.^6, 7, 8, 9, 10^ A direct comparison of the prevalence data between sexes in different studies has been hampered by the differences in the population sampling strategy, the sensitivity of the HPV genotyping assay and the number of genotypes detected. Thus, less is known about the sex-dependent differences of anogenital HPV infection characteristics. Only one study reported that HPV prevalence was lower in the cervical samples of women than in the penile samples of men (36.7% vs 50.8%, respectively) in an HIV-negative population in South Africa, but the sample size was relatively small.^11^

The aim of this study was to define the prevalence and type distribution of HPV in the general population of women and men in southern China using the same protocol for HPV genotyping. The whole picture of HPV distribution and the risk factors in both sexes would be helpful for developing more effective preventive policies for HPV infection.

## MATERIALS AND METHODS

### Study population

A population-based study among women and men was conducted in a rural site and an urban site of Liuzhou city, Guangxi, China, from March to July 2014. Participants were eligible if they were 18–55 years old, had ever been involved in sexual activity at enrollment, and were willing to refrain from sexual activity (including vaginal penetration, anal penetration, or any genital contact) and to avoid washing the genitals for 48 h before sample collection. Exclusion criteria consisted of having received an HPV vaccine, having reported serious illnesses or current pregnancy for women. Most of the volunteers were recruited from the general population through media advertising, including local newspapers and TV news, and educational presentations in communities. In addition, to increase the enrollment of younger people, students from Guangxi University of Science and Technology were recruited through flyers and posters.

Before study initiation, the Ethics Committee of Liuzhou Centers for Disease Controls approved all of the study procedures. At the enrollment visit, all of the participants gave written informed consent and underwent a clinical examination. Each participant was interviewed individually by a trained interviewer to complete a questionnaire. Then, genital exfoliated cell samples were obtained from each participant.

### Specimen collection

For women, three iCleanhcy flocked swabs (Huachenyang Corporation, Shenzhen, China) were used to collect specimens from the VU, VA and perianal/anal (PA) canal. Physicians used a swab to swipe the region from the clitoris to the labia minora and the labia majora in a zigzag pattern to ensure acquisition of the exfoliated cells. A second swab was used to collect a sample from the lower 1/3 of the VA by separating the labia of the VU. Then, a single-use speculum was inserted into the VA, and exfoliated cells from the upper 1/3 of the VA were obtained. A third swab was used to sweep 360° around the perianal area and was then inserted into the anal canal to obtain exfoliated cells.

For men, two pre-wetted saline iCleanhcy flocked swabs (Huachenyang Corporation, Shenzhen, China) were used to collect specimens from the penis/glans penis/coronary sulcus (PGC) and PA canal. Physicians used a swab to rub up and down the entire skin surface of each of the quadrants of the penis shaft. Then, the same swab was swept 360° around the glans penis and coronary sulcus. For uncircumcised men, exfoliated cells of the foreskin were collected using the same swab. The cells of the perianal and anal canal were obtained using the second swab in the same way as for women.

The swab samples were placed into separate vials containing 1 ml of PreservCyt solution (Hologic Corporation, Marlborough, MA, USA), and the tubes containing the specimens were stored at room temperature until they were transported to the laboratory of Zeesan Biotech Corporation, Xiamen, China, within two weeks for detection and typing of HPV DNA.

### HPV DNA testing

HPV testing of the swab specimens was conducted using a multicolor real-time PCR and melting curve analysis. GP5+/6+ was used as the general primer rather than the MY09/MY11 or SPF10 primers, which amplified longer or shorter target L1 fragments, respectively.^12, 13, 14^ DNA extraction was performed using the Lad-Aid 824 system (Zeesan Biotech Corporation, Xiamen, China) according to the instructions of the manufacturer. In brief, 200 μL aliquots of the clinical material were digested with 3 M guanidine hydrochloride solution for 300 s. The DNA was eluted in 150 μL of 10 mmol/L Tris-HCl buffer (pH 7.5) at room temperature. The DNA was stored at −80 °C until use.

The specimens were tested for the presence of HPV by amplifying 5 μL of the DNA extracts with the GP5+/6+ L1 consensus primer system and Taq HS polymerase (Takara Biotechnology Corporation, Dalian, China). Each 25 μL amplification reaction contained 75 mmol/L Tris-HCl (pH 8.0), 20 mmol/L (NH4)_2_SO_4_, 4 mmol/L MgCl_2_, 0.01% (vol/vol) Tween 20, 1 unit of Taq HS DNA polymerase, 200 μmol/L of each deoxynucleoside triphosphate, 100 nmol/L primer GP5+, 1 μmol/L primer GP6+, 100 nmol/L primer IPC-F, 1 μmol/L primer IPC-R, and 250 nmol/L of each of the 16 HPV and glyceraldehyde-3-phosphate dehydrogenase (GAPDH) probes, which were used to determine the adequacy of specimens. For every PCR plate, a negative control (H_2_O) and a positive control (containing HPV 16-, HPV 45- and HPV 58-positive plasmids) were run to control for possible contamination and accuracy. The samples were amplified using an SLAN-96p real-time PCR instrument. The following amplification profile was used: 50 °C 5 min→95 °C 3 min→(95 °C 15 s→50 °C 20 s (−1 °C per cycle)→78 °C 20 s) for 10 cycles→(95 °C 15 s→56 °C 16 s→78 °C 20 s) for 50 cycles. HPV genotyping was conducted based on the specific melting temperature of the product produced by hybridization of the PCR products and fluorescent probes. The procedure was set up as follows: 95 °C 1 min→35 °C 3 min→40 °C ~85 °C (at a heating rate of 0.04 °C /s).

### Definition of outcomes

Thirteen HPV types are labeled oncogenic types, including 12 types (HPV 16, 18, 31, 33, 35, 39, 45, 51, 52, 56, 58, and 59) that are carcinogenic to humans and HPV 68, which is probably carcinogenic to humans. In addition, HPV types 6, 11 and 66 were also detected in our study. HPV 66 is possibly carcinogenic to humans, and HPV 6 and 11 are not carcinogenic to humans.^15^ A specimen was deemed adequate if the GAPDH or HPV genotyping was positive. The subject having at least one adequate specimen was included in the analysis. The presence of any HPV DNA in the VA, VU or PA for women or in the PGC or PA for men was defined as a positive result. The absence of any HPV DNA from the VA, VU and PA for women or in the PGC and PA for men was defined as a negative result. Subjects without adequate samples were excluded from the analysis. The classification of any HPV or oncogenic HPV was defined as a positive test result for at least one of the 16 tested HPV types or at least one of 13 oncogenic HPV types.

### Statistical analyses

The data were analyzed using SAS version 9.4 (SAS Institute, Cary, NC, USA), and *P*<0.05 was considered statistically significant. The HPV prevalence in women and men was estimated for any, oncogenic and specific HPV type. The HPV prevalence was compared using Pearson's *χ*^2^-test, correction for the *χ*^2^-test, or the Fisher exact test. The 95% confidence intervals (CIs) for prevalence were calculated by the binomial exact test.

Bivariate analysis and multivariate logistic regression were conducted to assess the odds ratios (ORs) and 95% CIs, respectively, for oncogenic HPV and HPV 6/11 infection factors. Variables with a *P*-value<0.20 in the bivariate logistic analysis were included in the multivariate logistics model using stepwise selection, with a 0.05 significance level for entry into the model and 0.05 for removal.

## RESULTS

A total of 4687 participants, including 2378 women and 2309 men, aged 18–55 years were enrolled. After removing the participants without adequate samples, 2344 (98.6%) women and 1937 (83.9%) men were included in the analysis. Table 1 shows the distribution of the sociodemographic and sexual behavioral characteristics of the volunteers in the analysis by sex. The median ages of both women and men were 38 years. Most of the participants had a low education level and low income and were married or cohabitating. The majority had stayed in a hotel, and a larger percentage of men than women had used a towel supplied by the hotel (62.2% vs 37.5%, respectively, *P<*0.0001). Overall, the majority of the participants reported their age at first sexual intercourse was 18–25 years. At enrollment, most (56.2%) of the participants had one lifetime sex partner (LSP), 30.4% had two to three LSPs and 13.3% had four or more LSPs. Women were more conservative than men, having fewer LSPs and sexual partners in the year before the study (*P<*0.0001). The percent of women reporting a history of sexually transmitted diseases (STDs) was more than that of men (16.7% vs 7.2%, respectively, *P<*0.0001).

The overall prevalence of any HPV infection in the study population was 15.4% (95% CI: 14.4–16.6). Statistically significant differences were observed by sex for the prevalence of any tested HPV (19.5% for women vs 10.5% for men, *P<*0.001), any oncogenic type (18.7% for women vs 9.4% for men, *P<*0.001), HPV 16/18 (4.6% for women vs 1.5% for men, *P<*0.001), HPV 6/11/16/18 (5.6% for women vs 2.6% for men, *P<*0.001) and HPV 6/11/16/18/31/33/45/52/58 (15.2% for women vs 8.1% for men, *P<*0.001, Table 2); however, the prevalence of HPV 6/11 infection in women and men was not significantly different (1.4% for women vs 1.2% for men, *P=*0.6832).

**Figure 1:**
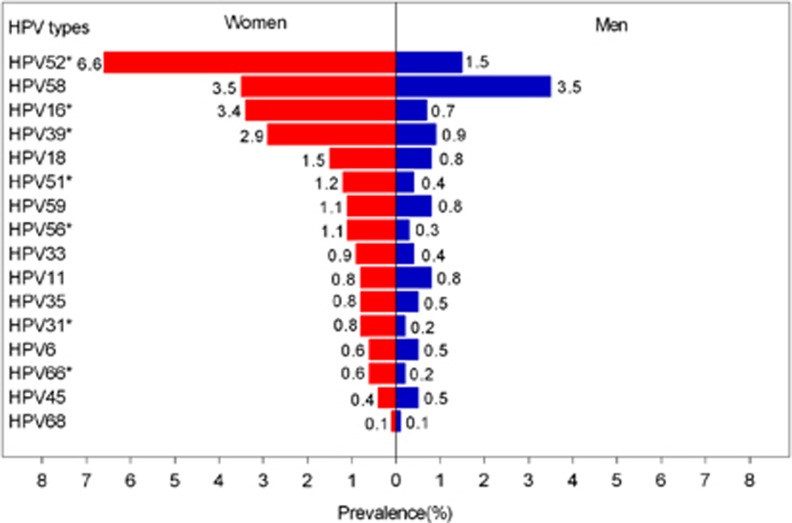
Type special HPV infection distribution in women and men at study enrollment. *Significant differences between women and men at *P* <0.05 with Pearson's *χ*^2^-test, correction for the *χ*^2^-test or the Fisher exact test. Human papillomavirus, HPV.

In both sexes, HPV 52 (4.3%), HPV 58 (3.5%) and HPV 16 (2.2%) were the most commonly detected infections, followed by HPV 39 (2.0%) and HPV18 (1.2%). The prevalence of any other HPV type was <1.0%. For women, the three most common oncogenic HPV types were HPV 52 (6.6%), HPV 58 (3.5%) and HPV 16 (3.4%), whereas in men, HPV 58 (3.5%), HPV 52 (1.5%) and HPV 39 (0.9%) were the most common (Figure 1).

**Figure 2:**
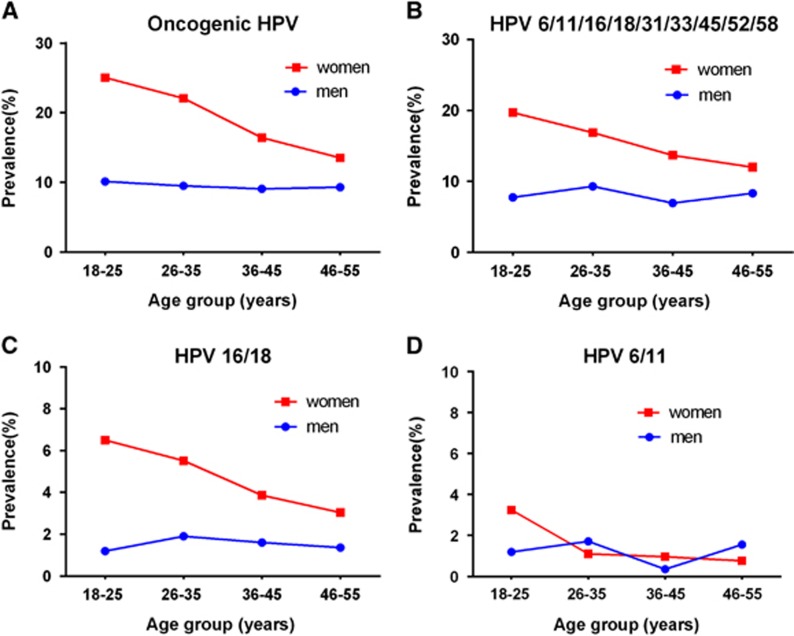
Age-specific HPV prevalence at enrollment in women and men. (**A**) Oncogenic HPV infection; (**B**) HPV 6, 11, 16, 18, 31, 33, 45, 52 or 58 infection; (**C**) HPV 16 or 18 infection; (**D**) HPV 6 or 11 infection. Human papillomavirus, HPV.

Figure 2 shows the age-specific HPV prevalence in women and men, stratified by different HPV types. For any group, the HPV prevalence was highest in women aged 18–25 years and then continuously declined with age. For the oncogenic HPV types, the prevalence peaked between 18 and 25 years old (25.1%) and then generally decreased and reached 13.5% between 46 and 55 years old. In contrast, the prevalence of all the HPV groups in men was unrelated to age (all *P*>0.05).

Table 3 presents the risk factors for oncogenic HPV and HPV 6/11 infection by sex based on the univariate and multivariate logistic regression models. In the multivariate analyses, two sexual behavior factors, LSPs (in comparison with one partner: OR, 1.5 (95% CI 1.2–1.9) for two to three partners and OR, 2.2 (95% CI 1.4–3.6) for more than or equal to four partners) and number of sexual partners in the past year (in comparison with zero to one partner: OR, 1.6 (95% CI 1.1–2.6) for more than or equal to two partners), were associated with oncogenic HPV in women (Table 3). In addition, living in an urban area, being single/divorced/separated/widowed and having used a towel supplied by a hotel were independently associated with oncogenic HPV in women. For HPV 6/11 infection in women, only two sexual behavior factors, LSPs and number of sexual partners in the past year, were significant. For men, having used a towel supplied by a hotel (OR, 1.5 (95% CI 1.1–2.0)) and their sexual partners ever having sex with others (OR, 7.4 (95% CI 1.5–37.0)) were independently associated with oncogenic HPV and HPV 6/11 infection, respectively, in the multivariate analyses.

## DISCUSSION

To our knowledge, this is the first study to compare the prevalence of anogenital HPV and associated risk factors between sexes in the same community in China. The standardized clinical, sampling and laboratory methods allow a direct comparison of HPV DNA prevalence and risk factor data across both sexes. The data indicate that the prevalence of any oncogenic HPV type in women is higher than in men, whereas the prevalence of HPV 6/11 is comparable between the sexes. HPV infection peaks in women aged 18–25 years and then decreases slowly, whereas the prevalence remains stable among all age groups in men. In addition to sexual behavior, health habits, such as using a towel supplied by a hotel, are significantly associated with HPV infection in both women and men.

There is a paucity of studies on the prevalence of HPV in males in China. We observed a lower prevalence of HPV infection (10.5%) in men than in the only other study we are aware of, which reported the HPV prevalence of 40 types (17.8%) in males in Henan province, north China.^16^ In the current study, we assessed only 16 HPV types, including 13 high- and 3 low-risk types. For carcinogenic HPV types, the estimated prevalence is 9.4%, which is higher than in the Henan study (6.4%). The discrepancy might be explained by the different anatomic sampling sites and the different HPV typing techniques. On the other hand, the sexual behavior characteristics differ between north and south China. Most (87.6%) of the participants in Henan reported having zero to one lifetime sexual partner, whereas the proportion with zero to one partner in our study was only 45.0%. The number of lifetime sexual partners is significantly associated with increased HPV prevalence in men.^17, 18, 19^ A higher rate of any tested HPV (21.2%) was reported among men on five continents, although a similar number of HPV types was assessed.^17^ Among the five continents, the participants in Asia/Pacific had the lowest HPV DNA prevalence. The reported prevalence of total and high-risk HPV, respectively, was much higher in men from the United States (61.3%, 23.3%), Mexico (61.9%, 30.4%) and Brazil (72.3%, 36.1%), likely because of sexual behavior—the average reported LSPs of the participating men were much higher than in the current study.^9^

For the women in our study, the prevalence of oncogenic HPV infection (18.7%) was similar to the rate reported by a pooled study that included a total of 30 207 women (17.7%) in 17 studies throughout China, and the young women aged 18–25 years (15.3%) in a large phase III clinical trial in Jiangsu, China; however, these studies collected samples from the cervix, in contrast with the three anatomic (VU, VA and PA) sites used in our study.^7, 20^

Few studies enroll both women and men from the same population; therefore, a direct comparison of the prevalence of HPV in women and men is difficult due to the methodological differences among studies. A study from South Africa reported that the prevalence of carcinogenic HPV was similar in HIV-negative men (22.4%) and HIV-negative women (22.7%), but was higher for non-carcinogenic HPV in men (33.2%) than in women (14.0%).^11^ In the current study, samples from more anatomic sites were assessed, and the data showed that the estimated prevalence of carcinogenic HPV was higher in women than in men. A potential explanation for this phenomenon might be the different physiological structures, and therefore compared with men, women may have a higher incidence rate or lower clearance rate.^21^ A Dutch study found that persistent infection was observed more frequently in women than in men (OR=3.88).^22^ Further prospective studies should be conducted to illustrate the differences in HPV natural history in both sexes.

The five most dominant circulating high-risk HPV types were HPV 52, 58, 16, 39 and 18, which is concordant in the women and men in the current study, although the order is slightly different between the sexes. Epidemiological studies have found that type-specific HPV infection distribution is different among world regions. Our finding that HPV 52, 58 and 16 were the most frequent oncogenic HPV types in women was concordant with the findings of previous studies in China and different from an American study (HPV 53, 16 and 51) and a meta-analysis of one million women on five continents (HPV 16, 18 and 52).^20, 23, 24, 25, 26, 27^ In men, the three most common types were HPV 58, 52 and 39, which was different from other studies in Brazil (HPV 16, 66 and 59), Mexico (HPV 59, 51 and 16), the United States (HPV 16, 51 and 66), Anyang, China (HPV 16, 18 and 58), and on five continents (HPV 51, 16 and 56).^9, 16, 17^ In both women and men, HPV 52 and 58 were the most prevalent types in our study, which further implies the necessity to vaccinate against HPV 52 and 58 in China.

In agreement with most studies, we found that age does not influence genital HPV prevalence in men.^9^ For women, the age trend of HPV infection differed by region.^28^ In general, the rate peaks in the years after sexual debut (<25 years old), is then relatively steady across age, or declines in older ages, or is characterized by a U shape with a second peak during older age (Central and South America, and Africa). In China, Zhao *et al.* reported that age-stratified high-risk HPV infection in women presented a U shape, which was different from our finding of a downward trend as age increased. Whether this difference is due to different anatomic sampling sites is not clear.^7^

For non-oncogenic HPV infection types 6 and 11, sexual behavior, such as LSPs and/or the number of sex partners in the past year, played a statistically significant role among men and women. This finding mirrors those observed in other studies.^17, 26, 29, 30^ We also found an association of ever having used a towel supplied by a hotel with oncogenic HPV infection in both sexes, which may provide new information for the secondary prevention of oncogenic HPV infection among women and men. No other questionnaire-based information, including age at first sexual intercourse, condom use or STDs, had a significant association with HPV infection.

Limitations need to be considered for this study. First, compared with women, the lower proportion of GAPDH in men, which may be partially caused by the sampling method and the difference in genital structure, may result in an underestimate of HPV prevalence in men; however, we believe this possibility is quite low because there was no significant difference when we analyzed a subset of men who had adequate specimens in all the anatomic sites (data not shown). Second, some studies indicated that HPV detection at the scrotum (20%–34%) was higher than the perianal area (14%–20%) and anal canal (11%–17%).^31, 32^ Our study did not collect exfoliated cells from the scrotum, which may have resulted in a lower detected prevalence in men. Third, we tested for only 16 HPV types, which excluded data on the prevalence of additional non-oncogenic HPV types.

In conclusion, this preliminary study highlights a higher burden of oncogenic HPV infection in generally healthy women compared with men in China, whereas the prevalence of HPV 6 and 11 is similar between the sexes. The data indicate that the interaction of host and virus might be different among high- and low-risk HPV types.

## Figures and Tables

**Table 1:** Sociodemographic and sexual behavioral characteristics of the study participants by sex

**Factor**	**Women (*n*=2344)**	**Men (*n*=1937)**	**Total (*n*=4281)**
	***n* (%)**	***n* (%)**	***n* (%)**
*Age (years)*			
18–25	431 (18.4)	335 (17.3)	766 (17.9)
26–35	634 (27.0)	526 (27.2)	1160 (27.1)
36–45	621 (26.5)	561 (29.0)	1182 (27.6)
46–55	658 (28.1)	515 (26.6)	1173 (27.4)
Median (*Q*)	38 (28, 46)	38 (29, 46)	38 (28, 46)
*Site*			
Rural	953 (40.7)	787 (40.6)	1740 (40.6)
Urban	1391 (59.3)	1150 (59.4)	2541 (59.4)
*Education (years)*			
<12	1937 (82.6)	1557 (80.4)	3494 (81.6)
≥12	407 (17.4)	380 (19.6)	787 (18.4)
*Annual household income (CNY)*			
<50000	1874 (79.9)	1463 (75.5)	3337 (77.9)
≥50000	470 (20.1)	474 (24.5)	944 (22.1)
*Marital status*			
Married/cohabitating	1946 (83.0)	1458 (75.3)	3404 (79.5)
Single/divorced/separated/widowed	398 (17.0)	479 (24.7)	877 (20.5)
*Ever used a sauna*			
No	1722 (73.5)	1322 (68.2)	3044 (71.1)
Yes	622 (26.5)	615 (31.8)	1237 (28.9)
*Ever used a towel supplied by the sauna*			
No	1879 (80.2)	1413 (72.9)	3292 (76.9)
Yes	465 (19.8)	524 (27.1)	989 (23.1)
*Ever stayed in a hotel*			
No	806 (34.4)	339 (17.5)	1145 (26.7)
Yes	1538 (65.6)	1598 (82.5)	3136 (73.3)
*Ever used a towel supplied by the hotel*			
No	1466 (62.5)	733 (37.8)	2199 (51.4)
Yes	878 (37.5)	1204 (62.2)	2082 (48.6)
*Age at the time of first sexual intercourse (years)*			
<18	139 (5.9)	161 (8.3)	300 (7.0)
18–25	1971 (84.1)	1475 (76.1)	3446 (80.5)
>25	234 (10.0)	301 (15.5)	535 (12.5)
Median (*Q*)	21 (20, 23)	22 (19, 24)	21 (20, 24)
*Lifetime number of sex partners*			
1	1536 (65.5)	871 (45.0)	2407 (56.2)
2–3	711 (30.3)	592 (30.6)	1303 (30.4)
≥4	97 (4.1)	474 (24.5)	571 (13.3)
Median (*Q*)	1 (1, 2)	2 (1, 3)	1 (1, 3)
*Number of sex partners in past year*			
0–1	2236 (95.4)	1635 (84.4)	3871 (90.4)
>1	108 (4.6)	302 (15.6)	410 (9.6)
Median (*Q*)	1 (1, 1)	1 (1, 2)	1 (1, 1)
*Sex partners having sex with others*			
No	970 (41.4)	687 (35.5)	1657 (38.7)
Yes	108 (4.6)	96 (5.0)	204 (4.8)
Unknown	1266 (54.0)	1154 (59.6)	2420 (56.5)
*Previous STDs diagnosis*			
No	1952 (83.3)	1797 (92.8)	3749 (87.6)
Yes	392 (16.7)	140 (7.2)	532 (12.4)
*Frequency of condom use*			
Never	1265 (54.0)	934 (48.2)	2199 (51.4)
Sometimes	951 (40.6)	843 (43.5)	1794 (41.9)
Always	128 (5.5)	160 (8.3)	288 (6.7)

Abbreviations: Chinese Yuan, CNY; inter-quartile range, *Q*; sexually transmitted diseases, STDs.

**Table 2:** Grouped HPV-type distribution by sex

**Grouped HPV types**	**Women (*n*=2344)**		**Men (*n*=1937)**		***P*-value**^a^
	***n***	**% (95% CI)**	***n***	**% (95% CI)**	
Any HPV^b^	457	19.5 (17.9, 21.2)	204	10.5 (9.2, 12.0)	<0.0001
Any oncogenic HPV^c^	439	18.7 (17.2, 20.4)	183	9.4 (8.2, 10.9)	<0.0001
HPV 6 or 11	32	1.4 (0.9, 1.9)	23	1.2 (0.7, 1.8)	0.6832
HPV 16 or 18	107	4.6 (3.8, 5.5)	30	1.5 (1.1, 2.2)	<0.0001
HPV 6, 11, 16 or 18	131	5.6 (4.7, 6.6)	51	2.6 (1.97, 3.5)	<0.0001
HPV 6, 11, 16, 18, 31, 33, 45, 52 or 58	356	15.2 (13.8, 16.7)	157	8.1 (6.9, 9.4)	<0.0001

Abbreviations: confidence interval, CI; human papillomavirus, HPV.

a Significant difference between men and women at *P*<0.05 with Pearson's *χ*^2^-test.

b Any HPV type in this study included HPV 6, 11, 16, 18, 31, 33, 35, 39, 45, 51, 52, 56, 58, 59, 66 and 68.

c Any oncogenic HPV type in this study included HPV 16, 18, 31, 33, 35, 39, 45, 51, 52, 56, 58, 59 and 68.

**Table 3:** Univariate and multivariate analyses of factors associated with oncogenic HPV and HPV 6/11 infection by sex

**Factor**	**Oncogenic HPV infection in women**		**HPV 6/11 infection in women**		**Oncogenic HPV infection in men**		**HPV 6/11 infection in men**	
	**OR (95% CI)**	**AOR (95% CI)**^a^	**OR (95%CI)**	**AOR (95%CI)**^b^	**OR (95% CI)**	**AOR (95% CI)**^c^	**OR (95% CI)**	**AOR (95% CI)**^d^
*Age (years)*								
18–25	1.0		1.0		1.0		1.0	
26–35	0.9 (0.6, 1.1)		0.3 (0.1, 0.8)		0.9 (0.6, 1.5)		1.4 (0.4, 4.7)	
36–45	0.6 (0.4, 0.8)		0.3 (0.1, 0.8)		0.9 (0.6, 1.4)		0.3 (0.1, 1.6)	
46–55	0.5 (0.3, 0.6)		0.2 (0.1, 0.6)		0.9 (0.6, 1.5)		1.3 (0.4, 4.4)	
*Site*								
Rural	1.0	1.0	1.0		1.0		1.0	
Urban	2.1 (1.7, 2.7)	1.7 (1.3, 2.2)	2.1 (0.9, 4.6)		0.8 (0.6, 1.1)		0.9 (0.4, 2.0)	
*Education (years)*								
<12	1.0		1.0		1.0		1.0	
≥12	1.2 (0.9, 1.5)		0.9 (0.3, 2.3)		0.8 (0.5, 1.1)		0.2 (0.1, 1.4)	
*Annual household income (CNY)*								
<50000	1.0		1.0		1.0		1.0	
≥50000	1.2 (0.9, 1.5)		0.7 (0.3, 1.9)		1.1 (0.8, 1.5)		2.0 (0.9, 4.7)	
*Marital status*								
Married/cohabitating	1.0	1.0	1.0		1.0		1.0	
Single/divorced/separated/widowed	2.2 (1.7, 2.8)	1.4 (1.1, 1.9)	3.4 (1.7, 7.0)		0.9 (0.6, 1.3)		1.6 (0.7, 3.9)	
*Ever used a sauna*								
No	1.0		1.0		1.0		1.0	
Yes	1.8 (1.4, 2.2)		2.2 (1.1, 4.4)		1.1 (0.8, 1.5)		1.5 (0.5, 2.7)	
*Ever used a towel supplied by the sauna*								
No	1.0		1.0		1.0		1.0	
Yes	2.0 (1.6, 2.6)		1.9 (0.9, 4.0)		1.1 (0.8, 1.6)		1.5 (0.6, 3.4)	
*Ever stayed in a hotel*								
No	1.0		1.0		1.0		1.0	
Yes	1.5 (1.2, 1.9)		1.9 (0.8, 4.4)		1.0 (0.7, 1.4)		2.2 (0.5, 9.6)	
*Ever used a towel supplied by the hotel*								
No	1.0	1.0	1.0		1.0	1.0	1.0	
Yes	2.0 (1.6, 2.4)	1.3 (1.1, 1.7)	1.9 (0.9, 3.8)		1.5 (1.1, 2.0)	1.5 (1.1,2.0)	2.9 (1.0, 8.6)	
*Age at the time of first sexual intercourse (years)*								
<18	1.0		1.0		1.0		1.0	
18–25	0.5 (0.4, 0.8)		0.2 (0.1, 0.5)		1.3 (0.7, 2.3)		0.7 (0.2, 2.2)	
>25	0.4 (0.3, 0.7)		0.3 (0.1, 0.9)		1.0 (0.5, 2.0)		0.4 (0.1, 2.1)	
*Lifetime number of sex partners*								
1	1.0	1.0	1.0	1.0	1.0		1.0	
2–3	1.9 (1.5, 2.4)	1.5 (1.2, 1.9)	4.7 (2.1, 10.4)	3.5 (1.5, 8.2)	1.2 (0.8, 1.7)		1.3 (0.4, 3.8)	
≥4	4.1 (2.7, 6.3)	2.2 (1.4, 3.6)	7.3 (2.2, 24.2)	2.9 (0.7, 11.7)	1.4 (1.0, 2.0)		2.7 (1.1, 7.0)	
*Number of sex partners in past year*								
0–1	1.0	1.0	1.0	1.0	1.0		1.0	
≥2	3.3 (2.2, 5.0)	1.6 (1.1, 2.6)	8.8 (3.9, 19.4)	4.5 (1.7, 11.8)	1.5 (1.1, 2.2)		1.9 (0.8, 4.9)	
*Sexual partners having sex with others*								
No	1.0		1.0		1.0		1.0	1.0
Yes	1.7 (1.1, 2.6)		0.7 (0.1, 5.3)		1.8 (0.9, 3.5)		7.4 (1.5, 37.0)	7.4 (1.5, 37.0)
Unknown	0.9 (0.7, 1.1)		1.1 (0.5, 2.2)		1.3 (0.9, 1.8)		3.4 (1.0, 11.7)	3.4 (1.0, 11.7)
*Previous STDs diagnosis*								
No	1.0		1.0		1.0		1.0	
Yes	1.7 (1.4, 2.3)		1.4 (0.6, 3.3)		1.1 (0.6, 1.9)		2.8 (0.9, 8.2)	
*Frequency of condom use*								
Never	1.0		1.0		1.0		1.0	
Sometimes	1.6 (1.3, 2.0)		1.6 (0.8, 3.3)		0.9 (0.6, 1.2)		2.1 (0.9, 5.0)	
Always	1.6 (1.1, 2.5)		0.7 (0.1, 5.4)		0.9 (0.5, 1.5)			

Abbreviations: adjusted odds ratio, AOR; confidence interval, CI; Chinese Yuan, CNY; human papillomavirus, HPV; lifetime sex partner, LSP; odds ratio, OR; sexually transmitted diseases, STDs.

a Age, region, marital status, ever used a sauna, ever used a towel provided by the sauna, ever stayed at a hotel, ever used a towel provided by a hotel, age at the time of first sexual intercourse, LSPs, sex partners in past year, condom use, sexual partners having sex with others and previous STD diagnosis remained in the multivariate regression model.

b Age, region, marital status, ever used a towel provided by the sauna, ever stayed at a hotel, ever used a towel at the hotel, age at time of first sexual intercourse, LSPs and sex partners in past year remained in the multivariate regression model.

c Region, education, ever used a towel provided by the hotel, LSPs, sex partners in past year and sexual partners having sex with others remained in the multivariate regression model.

d Education, income, ever used a towel provided by the hotel, LSPs, sex partners in past year and sexual partners having sex with others remained in the multivariate regression model.

ORs and 95% CIs derived by univariate logistic regression analysis; AORs and 95% CIs derived by multivariate logistic regression models with stepwise selection.
